# Kidney Health Biomarkers and Estimated Glomerular Filtration Rate Among Indonesian Tofu Production Workers: A Pilot Cross‐Sectional Study

**DOI:** 10.1002/hsr2.73431

**Published:** 2026-10-28

**Authors:** Putri Ayuni Alayyannur, Yustinus Denny Ardyanto Wahyudiono, Addien Nadira, Ritwan Agus Prasetyo, Edza Aria Wikurendra

**Affiliations:** ^1^ Department of Occupational Safety and Health Faculty of Public Health, Universitas Airlangga Surabaya Indonesia; ^2^ Occupational Health and Safety Management Research Group, Faculty of Public Health Universitas Airlangga Surabaya Indonesia; ^3^ Master Programme in Occupational Health and Safety, Faculty of Public Health Universitas Airlangga Surabaya Indonesia; ^4^ Department of Health Sciences Faculty of Humanities and Health Sciences, Curtin University Malaysia Miri Malaysia; ^5^ Research Fellow of Occupational Safety and Health Department, Faculty of Public Health Universitas Airlangga Surabaya Indonesia

**Keywords:** biomonitoring, food industry workers, glomerular filtration rate, kidney injury molecule‐1, neutrophil gelatinase‐associated lipocalin, occupational health

## Abstract

**Background and Aims:**

Small‐scale food‐production workers may experience heat strain, dehydration, and combustion‐related exposures, but evidence on early kidney injury in these settings remains limited. This pilot study aimed to describe serum and urinary kidney injury molecule‐1 (KIM‐1) and neutrophil gelatinase‐associated lipocalin (NGAL) profiles and to assess, exploratorily, whether these biomarkers discriminated an estimated glomerular filtration rate (eGFR)‐defined kidney‐function category among Indonesian tofu production workers.

**Methods:**

A cross‐sectional complete‐case analysis included 32 workers from small‐scale tofu production units in East Java, Indonesia. Serum creatinine, serum and urinary KIM‐1, and serum and urinary NGAL were measured using laboratory microplate‐based assays. eGFR was calculated using the CKD‐EPI creatinine equation; < 90 mL/min/1.73 m^2^ was a study‐defined exploratory category, not a CKD diagnosis. Descriptive statistics, Mann–Whitney *U* tests, Spearman correlations, and receiver operating characteristic analysis with 2000‐resample bootstrap 95% confidence intervals were performed.

**Results:**

Ten of 32 workers (31.3%) had eGFR < 90 mL/min/1.73 m^2^, and four of 32 (12.5%) had serum creatinine above the laboratory threshold. Median KIM‐1 and NGAL concentrations were lower in the eGFR < 90 group across all four biomarker measures. Discriminatory performance was limited, with area‐under‐the‐curve values ranging from 0.327 to 0.416. Urinary KIM‐1 and NGAL were analysed as absolute concentrations without urinary creatinine normalisation; hydration‐related variation in urine concentration may have increased measurement variability and weakened observed associations and discrimination. With 32 workers and 10 eGFR < 90 observations, the AUC estimates are imprecise and not definitive measures of diagnostic performance.

**Conclusion:**

KIM‐1 and NGAL showed limited discrimination of the study‐defined eGFR < 90 category; this result does not indicate poor overall utility as tubular injury biomarkers. Larger exposure‐informed studies should incorporate creatinine‐normalised urinary biomarkers, albuminuria, repeated kidney‐function measurements, and objective assessment of heat and nephrotoxic exposures.

## Key Points

- What is known
  ∘ Tubular injury biomarkers may provide information beyond serum creatinine, but their interpretation depends on biological matrix, urine dilution adjustment, reference standard, and study design.

- What is new
  ∘ Among 32 Indonesian tofu production workers, serum and urinary KIM‐1 and NGAL showed limited discrimination of the eGFR < 90 mL/min/1.73 m^2^ category.

- Clinical implications
  ∘ The findings are limited to discrimination of the study‐defined eGFR category and do not establish the overall utility of KIM‐1 or NGAL as tubular injury biomarkers.

## 1 Introduction

Chronic kidney disease (CKD) is an increasing global public health burden. The Global Burden of Disease 2023 analysis estimated that hundreds of millions of adults were living with CKD and that CKD remained a major contributor to mortality and disability worldwide [1]. In clinical and epidemiological practice, serum creatinine‐based eGFR remains central for kidney function assessment. eGFR primarily reflects filtration capacity and may be relatively insensitive to early tubular injury, particularly when renal reserve compensates for mild or localised damage [2, 3].

This limitation is especially relevant for occupational populations. Recent evidence has increasingly linked occupational kidney disease to heat stress, dehydration, heavy physical workload, heavy metals, organic solvents and other nephrotoxic exposures [4, 5, 6]. Small‐scale food production workers in low‐resource industrial settings may face overlapping risks, including hot work environments, irregular hydration and exposure to emissions from low‐temperature combustion of mixed waste or plastic‐containing materials. Open burning and uncontrolled combustion of plastic waste are recognised sources of toxic gases, particulate matter and potentially toxic elements, with implications for occupational and public health [7, 8].

In Indonesia, tofu is commonly produced in small‐scale or home‐based units. Boiling and heating processes may generate substantial heat exposure, while some production units use low‐cost waste‐derived fuels to reduce operational costs. Although these conditions suggest plausible kidney health risks, empirical data on early renal injury markers in tofu workers remain scarce. Workers may experience repeated subclinical renal stress before conventional markers become abnormal.

KIM‐1 and NGAL are among the most widely studied biomarkers of tubular injury. KIM‐1 is expressed in proximal tubular epithelial cells after toxic or ischaemic injury, whereas NGAL reflects inflammatory and tubular stress responses [9, 10]. Several studies have reported that these biomarkers may identify early kidney injury in exposed or high‐risk populations, although their performance depends strongly on the reference standard, urine normalisation method, disease phenotype and study design [10, 11]. Recent occupational evidence also indicates that KIM‐1 and NGAL may increase in exposed workers even when serum creatinine or eGFR remains within conventional limits [12].

Despite this biological plausibility, KIM‐1 and NGAL should not be assumed to be direct diagnostic markers of the study‐defined eGFR < 90 category. Tubular injury and glomerular filtration are related but non‐identical constructs; biomarker performance depends on the selected reference standard, timing of sample collection, urine dilution correction, exposure profile and disease phenotype. This distinction is important for occupational health studies, where biomonitoring may reveal early biological responses even when conventional clinical indices remain within reference ranges. The present study was designed as an exploratory occupational biomonitoring investigation among tofu production workers in Indonesia. The study aimed to describe serum and urinary KIM‐1 and NGAL profiles and assess their discriminatory performance across eGFR‐defined kidney function categories. The study was not designed to validate these biomarkers as stand‐alone diagnostic tests; it examined whether tubular injury biomarkers could provide complementary information for occupational kidney health assessment in an under‐studied worker population.

## 2 Methods

### 2.1 Study Design and Setting

This exploratory cross‐sectional occupational biomonitoring study was conducted among workers in small‐scale tofu production units in East Java, Indonesia. Here, “tofu factory” denotes small‐scale or home‐based production, not large mechanised facilities. These settings commonly involve wet soy processing, boiling/heating, manual handling and prolonged work in hot production areas. Kidney function and tubular injury biomarkers were assessed in this occupational setting. No objective environmental or biological exposure measurements were collected. Heat strain, dehydration and combustion‐related nephrotoxic pathways are discussed only as contextual hypotheses and not as causal exposure‐response relationships. Reporting followed the STROBE recommendations for cross‐sectional studies [13], which served as the primary reporting framework.

### 2.2 Participants and Sampling

Workers were eligible for inclusion when they were currently employed in tofu production, were present at the worksite during the field visit, were able to communicate during data collection, had worked in tofu production for at least 1 year, and voluntarily agreed to participate. Workers presenting with acute diarrhoea or fever at the time of data collection were excluded, as these conditions may alter hydration status and transiently influence serum creatinine, eGFR and urinary biomarker concentrations. Convenience sampling was applied in view of the absence of a complete sampling frame for tofu workers and the dependence of recruitment on worker availability and willingness during fieldwork. Each participant was assigned a unique sample identification code to link questionnaire, serum, and urine laboratory data while maintaining confidentiality. The final analytic dataset comprised participants with complete serum creatinine and biomarker data.

The final enroled cohort comprised 32 workers, and all 32 had complete serum creatinine and biomarker data and were retained in the complete‐case analysis. Separate counts for workers approached or screened, found ineligible, or declining participation were not available and are not reported. Given the pilot design and the absence of a complete sampling frame, recruitment was feasibility‐based, and no formal a priori power calculation was performed. A precision‐based sample‐size calculation was used to characterise the precision associated with the achieved sample. For a single proportion, *n* = *Z* ^2^ *p*(1 – *p*)/*d* ^2^. Under the conservative assumption *p* = 0.50 and Z = 1.96, a target precision of ±10 percentage points would require 97 participants. With *n* = 32, the maximum 95% margin of error is approximately ±17.3 percentage points. The achieved sample supports preliminary estimation and hypothesis generation, but not precise population estimates or stable diagnostic‐accuracy estimates.

### 2.3 Data Collection and Laboratory Measurements

Data collection consisted of a structured questionnaire and biological sample collection. The questionnaire was used to capture participant identifiers required for data linkage and basic worker information. Venous blood and spot urine samples were collected for serum creatinine, serum KIM‐1, urinary KIM‐1, serum NGAL, and urinary NGAL. Laboratory testing was performed by the Research Laboratory Installation, Universitas Airlangga Hospital. Serum creatinine was reported in mg/dL, KIM‐1 concentrations in pg/mL, and NGAL concentrations in ng/mL. Serum and urinary KIM‐1 were measured using the Human KIM‐1 ELISA kit (BioVendor, catalogue RBL006R; calibration range, 15.6–1000 pg/mL; limit of detection, 7.8 pg/mL). Manufacturer‐reported intra‐assay and inter‐assay coefficients of variation were 3.5% and 9.5%, respectively. Serum and urinary NGAL were measured using the Human Lipocalin‐2/NGAL Quantikine ELISA kit (R&D Systems, catalogue DLCN20; assay range, 0.156–10 ng/mL; sensitivity, 0.04 ng/mL). Manufacturer‐reported intra‐assay and inter‐assay coefficients of variation were 3.1%–4.4% and 5.6%–7.9%, respectively. KIM‐1 assays were read using endpoint absorbance at 450.0 nm and calculated from a quadratic calibration curve, whereas NGAL assays were read using endpoint absorbance at 450.0 nm and calculated from a four‐parameter calibration curve.

Instrument reports listed one well for each participant sample; sample‐level standard‐deviation fields and coefficients of variation were not populated. Technical duplicate measurements were not documented, and the statistical dataset used the reported concentration for each sample. Laboratory‐specific intra‐assay and inter‐assay coefficients of variation were not provided and were not calculated post hoc. Calibration‐curve fit was high for KIM‐1 (quadratic fit, *r* ^2^ = 1.000) and NGAL (four‐parameter fit, *r* ^2^ = 0.991); these statistics describe calibration performance and were not used as measures of assay precision. Urinary KIM‐1 and urinary NGAL were analysed as absolute urinary concentrations without urinary creatinine normalisation, as urinary creatinine was not measured. Hydration‐related variation in urine concentration may have increased within‐ and between‐participant variability and affected observed associations and discriminatory performance.

### 2.4 Kidney Function Assessment

eGFR was calculated from serum creatinine, age, and sex using the 2021 CKD‐EPI creatinine equation without a race coefficient [2]. For exploratory stratification, eGFR < 90 mL/min/1.73 m^2^ was treated as a study‐defined category of relatively lower filtration, not as a diagnosis of CKD or confirmed kidney impairment. CKD requires evidence of chronicity and/or markers of kidney damage; the present dataset did not include repeated kidney‐function assessment, albuminuria or urine albumin‐creatinine ratio, or cystatin C. The eGFR < 90 category represents only a lower single‐time‐point eGFR within this study.

### 2.5 Ethical Considerations

The study protocol was reviewed and approved by the Research and Public Health Service Ethics Committee, Faculty of Public Health, Universitas Indonesia (Ethical Clearance No. Ket‐550/UN2.F10.D11/PPM.00.02/2022). Written informed consent was obtained from all participants before data collection, following provision of information on the study objectives, biological sampling procedures, voluntary participation, and confidentiality. The study was conducted in accordance with the ethical principles of the Declaration of Helsinki.

### 2.6 Statistical Analysis

Analyses were conducted using a complete‐case dataset comprising workers with available serum creatinine, eGFR, and biomarker values. Data management, descriptive analysis, Mann–Whitney *U* tests, and Spearman rank correlation analyses were performed using IBM SPSS Statistics, version 26.0. ROC analysis, bootstrap 95% confidence intervals, and graphical visualisation were performed using R software, version 4.3.2, with the pROC package [14]. For biomarker variables with non‐normal distributions, median (IQR) was used as the primary descriptive summary. Participant characteristics were additionally described using mean (SD), median and range where clinically informative. Categorical variables were summarised as n (%) with the denominator stated in the table heading or text. Statistical notation was defined as follows: SD, standard deviation; IQR, interquartile range; ROC, receiver operating characteristic; AUC, area under the ROC curve; CI, confidence interval; *α*, a priori significance level; *ρ*, Spearman rank correlation coefficient; and *n*/*N*, numerator/denominator.

Biomarker distributions were compared between workers with eGFR ≥ 90 and < 90 mL/min/1.73 m^2^ using the Mann–Whitney *U* test, given the limited sample size and non‐normal distribution of biomarker concentrations. Spearman rank correlation was used to examine monotonic associations between each biomarker and eGFR. ROC analysis was performed to estimate the exploratory discriminatory performance of each biomarker against eGFR < 90 mL/min/1.73 m^2^. AUC estimates were reported with bootstrap 95% confidence intervals based on 2000 resamples. Sensitivity, specificity, and exploratory cut‐off values were derived using the maximum Youden index. All cut‐off values were interpreted as exploratory and not clinically validated.

A post hoc sensitivity analysis excluded Sample 35, whose serum creatinine concentration of 0.15 mg/dL was confirmed against the laboratory report, and repeated the ROC analysis using the same eGFR < 90 mL/min/1.73 m^2^ reference category to assess the influence of this extreme observation on the discriminatory estimates. Descriptive and non‐parametric procedures were reported with reference to standard biomedical statistical guidance [15]. No confirmatory hypothesis‐testing analysis was prespecified. Group comparisons by eGFR category, Spearman correlations, ROC analyses, and Youden‐index cut‐off estimation were treated as exploratory; no additional subgroup analyses were conducted. All inferential tests were two‐sided with an a priori significance level of *α* = 0.05. Given the exploratory pilot design and the evaluation of four biomarkers across related analyses, no adjustment for multiple comparisons was applied. *p* values were interpreted cautiously alongside the observed estimates and 95% confidence intervals where available.

Multivariable modelling was not performed, as the sample size and number of eGFR < 90 observations were insufficient to generate stable adjusted estimates. STARD principles supplemented STROBE for transparent reporting of the exploratory ROC component, particularly the index tests, reference‐standard limitations, missing data and statistical uncertainty [16]. Statistical reporting followed the SAMPL recommendations [17]. The statistical procedures comprised descriptive summaries, two‐sided Mann–Whitney U tests, two‐sided Spearman rank correlation tests, ROC analysis with 2000‐resample bootstrap 95% CIs, and maximum Youden index estimation.

## 3 Results

### 3.1 Participant Characteristics and Kidney Function Profile

A total of 32 workers had complete serum creatinine and biomarker data and were included in the complete‐case analysis. Participant characteristics and kidney function indicators are summarised in Table 1. The mean age was 42.0 years (SD 8.7; range 21‐61), and 20 workers (62.5%) were male. The mean serum creatinine concentration was 0.87 mg/dL (SD 0.23), with a median of 0.92 mg/dL. The mean eGFR was 100.1 mL/min/1.73 m2 (SD 23.7). Ten of 32 workers (31.3%) had eGFR < 90 mL/min/1.73 m2 and four of 32 workers (12.5%) had serum creatinine values above the laboratory threshold. The serum creatinine concentration of 0.15 mg/dL for Sample 35 was confirmed on the laboratory report; no alternative value or unit was listed. The value was retained in the primary analysis and evaluated in a sensitivity analysis excluding this participant.

**Table 1:** Participant characteristics and kidney function profile of tofu production workers.

Characteristic	Overall sample (*n* = 32)
Age, years, mean ± SD	42.0 ± 8.7
Age, years, median (range)	43.0 (21–61)
Male sex, *n* (%)	20 (62.5)
Female sex, *n* (%)	12 (37.5)
Serum creatinine, mg/dL, mean ± SD	0.87 ± 0.23
Serum creatinine, mg/dL, median (range)	0.92 (0.15–1.26)
eGFR, mL/min/1.73 m^2^, mean ± SD	100.1 ± 23.7
eGFR, mL/min/1.73 m^2^, median (range)	102.6 (54.4–179.9)
eGFR < 90 mL/min/1.73 m^2^, *n* (%)	10 (31.3)
Serum creatinine above laboratory threshold, *n* (%)	4 (12.5)

### 3.2 Biomarker Distribution

The distribution of kidney injury biomarkers is shown in Table 2. Median serum KIM‐1 was 102.0 pg/mL (IQR: 88.9–138.4), and median urinary KIM‐1 was 454.6 pg/mL (IQR: 284.4–882.3). Median serum NGAL was 0.617 ng/mL (IQR: 0.418–0.874), while median urinary NGAL was 4.64 ng/mL (IQR: 3.48–5.79). Substantial inter‐individual variability was observed across all biomarkers, particularly urinary KIM‐1. Owing to the unavailability of urinary creatinine measurements, urinary biomarker concentrations should be interpreted cautiously as unnormalised values.

**Table 2:** Distribution of serum and urinary kidney injury biomarkers.

Biomarker	Median (IQR)
Serum KIM‐1 (pg/mL)	102.0 (88.9–138.4)
Urinary KIM‐1 (pg/mL)	454.6 (284.4–882.3)
Serum NGAL (ng/mL)	0.617 (0.418–0.874)
Urinary NGAL (ng/mL)	4.64 (3.48–5.79)

### 3.3 Biomarker Comparison by eGFR Category

Biomarker comparisons by eGFR category are presented in Table 3. Median concentrations were lower in the eGFR < 90 group for all four biomarkers, with substantial overlap in the IQRs between groups. Two‐sided Mann–Whitney *U* test *p* values are reported with the corresponding group medians and IQRs in Table 3. These findings do not support a simple monotonic relationship between higher tubular injury biomarker concentrations and lower eGFR in this sample.

**Table 3:** Biomarker concentrations according to eGFR category; *p* values from two‐sided Mann–Whitney *U* tests.

Biomarker	eGFR ≥ 90 median (IQR)	eGFR < 90 median (IQR)	*p* value
Serum KIM‐1 (pg/mL)	109.5 (90.0–173.3)	99.4 (90.7–110.4)	0.46
Urinary KIM‐1 (pg/mL)	686.8 (314.1–944.7)	427.9 (283.8–609.3)	0.34
Serum NGAL (ng/mL)	0.760 (0.503–0.883)	0.472 (0.289–0.688)	0.13
Urinary NGAL (ng/mL)	4.81 (3.71–6.16)	4.47 (2.30–5.15)	0.28

### 3.4 Correlation With eGFR

Spearman correlation coefficients are presented in Table 4. All four coefficients were positive (*ρ* = 0.018–0.339), providing no evidence of an inverse association. Urinary KIM‐1 had the largest estimate (*ρ* = 0.339), while the remaining coefficients ranged from 0.018 to 0.190. Two‐sided *p* values are presented with the corresponding correlation coefficients in Table 4. These results further indicate that the biomarkers did not show the expected inverse relation with eGFR in this small cross‐sectional dataset.

**Table 4:** Spearman correlation between kidney injury biomarkers and eGFR; *p* values from two‐sided Spearman rank correlation tests.

Biomarker	Spearman rho with eGFR	*p* value
Serum KIM‐1	0.018	0.92
Urinary KIM‐1	0.339	0.06
Serum NGAL	0.145	0.43
Urinary NGAL	0.190	0.30

### 3.5 Exploratory ROC Analysis

Table 5 and Figure 1 present the exploratory ROC results. ROC analysis showed low discriminatory performance for all four biomarkers against eGFR < 90 mL/min/1.73 m2. All AUC values were below 0.5, with wide 95% confidence intervals indicating limited precision. Sensitivity was high at low cut‐off points for serum KIM‐1, urinary KIM‐1 and serum NGAL, but specificity was very low. These exploratory thresholds should not be used for clinical decision‐making or occupational screening without external validation. In the sensitivity analysis excluding Sample 35 (*n* = 31), the AUC point estimates were 0.436 for serum KIM‐1, 0.362 for urinary KIM‐1, 0.310 for serum NGAL, and 0.367 for urinary NGAL, compared with 0.416, 0.391, 0.327, and 0.377, respectively, in the primary analysis. All sensitivity‐analysis AUCs remained below 0.50; exclusion of the extreme creatinine observation did not materially change the exploratory interpretation.

**Table 5:** Exploratory ROC performance of biomarkers for eGFR < 90 mL/min/1.73 m^2^.

Biomarker	AUC (95% CI)	Exploratory cut‐off	Sensitivity, *n*/*N* (%)	Specificity, *n*/*N* (%)
Serum KIM‐1	0.416 (0.227–0.609)	84.94 pg/mL	10/10 (100%)	4/22 (18.2%)
Urinary KIM‐1	0.391 (0.190–0.609)	228.62 pg/mL	10/10 (100%)	1/22 (4.5%)
Serum NGAL	0.327 (0.126–0.563)	0.225 ng/mL	10/10 (100%)	1/22 (4.5%)
Urinary NGAL	0.377 (0.169–0.604)	4.42 ng/mL	6/10 (60.0%)	10/22 (45.5%)

**Figure 1:**
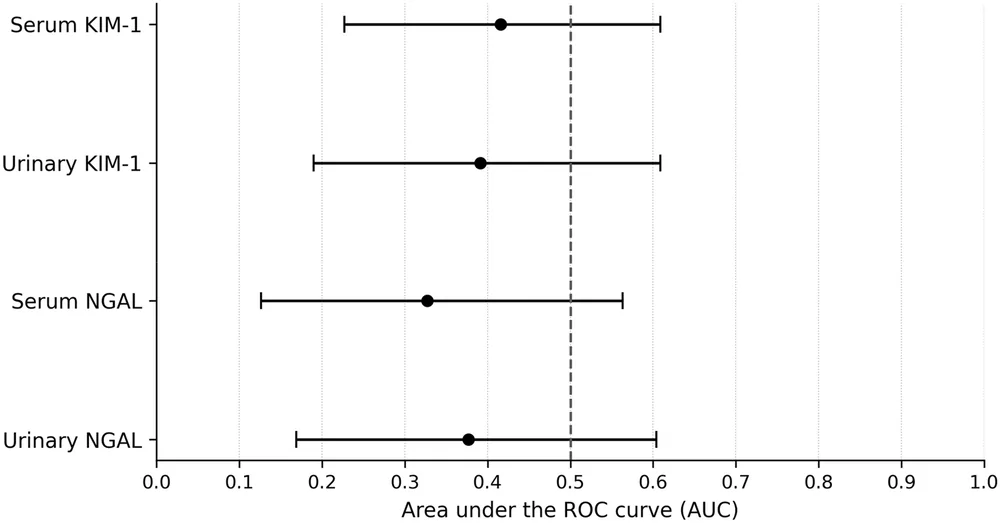
Exploratory AUC estimates and bootstrap 95% confidence intervals for serum and urinary KIM‐1 and NGAL against eGFR < 90 mL/min/1.73 m^2^. The dashed vertical line indicates AUC 0.50, representing no discrimination. All point estimates were below 0.50, and confidence intervals were wide, supporting cautious interpretation.

## 4 Discussion

This exploratory study examined serum and urinary KIM‐1 and NGAL among tofu production workers and assessed their performance against eGFR‐defined kidney function categories. Discrimination of eGFR < 90 mL/min/1.73 m2 was limited for all four biomarkers. Among the 10 of 32 participants with eGFR < 90 mL/min/1.73 m^2^, median concentrations were lower for all four biomarker measures, and AUC estimates ranged from 0.327 to 0.416 with wide 95% confidence intervals. With 32 participants and 10 eGFR < 90 observations, statistical power and precision were limited, and ROC/AUC estimates may be unstable. These preliminary AUC estimates do not establish that KIM‐1 or NGAL lack diagnostic value for kidney injury, particularly given the small sample and the eGFR‐based comparator.

The findings should be interpreted in light of biomarker biology. KIM‐1 and NGAL are primarily markers of tubular stress or injury, whereas eGFR is a functional measure of filtration. A weak relationship between tubular injury markers and eGFR is biologically plausible, particularly when kidney injury is early, heterogeneous, or compensated by renal reserve [9, 10, 18]. The low AUC values indicate limited discrimination of the study‐defined eGFR category in this sample. They should not be interpreted as evidence of poor overall utility of KIM‐1 or NGAL as kidney injury biomarkers, since tubular injury or stress and creatinine‐based glomerular filtration represent distinct biological constructs. For urinary biomarkers, lack of creatinine normalisation may have increased within‐ and between‐participant variability and attenuated associations with eGFR. This distinction is consistent with evidence that KIM‐1 may have diagnostic and prognostic relevance in kidney injury, while its interpretation depends on the kidney disease phenotype, biological matrix and selected comparator outcome [19].

The results contrast with studies in larger and more comprehensively characterised populations. For example, Ekanayake et al. [11] reported strong ROC performance for creatinine‐adjusted urinary KIM‐1 and NGAL in occupational groups in Sri Lanka, using comparator groups and urinary creatinine normalisation. Similarly, recent occupational toxicology evidence indicates that NGAL and KIM‐1 may increase among exposed workers despite conventional renal indices remaining within reference ranges [12]. The difference between those findings and the present study may reflect differences in sample size, population characteristics, control group availability, urine normalisation and the selected reference standard. Exposure‐related differences cannot be evaluated in this cohort, as exposure intensity was not measured.

Heat stress, dehydration and heavy workload are plausible occupational hypotheses supported by previous literature [5, 6, 20, 21, 22, 23]. No environmental or physiological monitoring of wet bulb globe temperature, core temperature, workload, fluid intake, urine specific gravity, or hydration status was performed in this cohort. These factors were not demonstrated in relation to the observed eGFR or biomarker patterns.

Combustion emissions are another literature‐supported occupational hypothesis. Open or low‐temperature combustion of plastic‐containing waste can release particulate matter, organic pollutants, and potentially toxic elements [4, 7, 8]. No environmental or biological exposure assessment of air pollutants, urinary metals, solvent metabolites, or other internal exposure markers was performed. Exposure presence, intensity, and individual uptake are unknown; no association with eGFR or biomarker values can be inferred.

The use of eGFR < 90 mL/min/1.73 m^2^ as the reference category warrants consideration. This threshold represented a study‐defined category of relatively lower filtration and was not a CKD definition. CKD requires evidence of chronicity and/or markers of kidney damage; repeated kidney‐function assessment, albuminuria, urine albumin–creatinine ratio, and cystatin C were unavailable. This single eGFR classification cannot establish CKD prevalence or confirmed kidney impairment. Interpretation of KIM‐1 and NGAL against this reference category should remain exploratory [3, 24].

The findings support a cautious, multimodal interpretation of occupational kidney health in this setting. Conventional kidney‐function measures, albuminuria, tubular biomarkers and repeated assessment should be considered together, while exposure‐related hypotheses require direct environmental or biological measurement. These findings can inform future studies incorporating direct exposure measurements.

### 4.1 Strengths and Limitations

A strength of this study is the paired assessment of serum and urinary tubular injury biomarkers alongside serum creatinine and eGFR in a rarely studied occupational group. Technical duplicate measurements and sample‐level, intra‐assay and inter‐assay coefficients of variation were not documented, so analytical precision could not be quantified. Several limitations should be considered. First, the convenience‐based sample comprised 32 participants, including 10 with eGFR < 90 mL/min/1.73 m^2^, limiting statistical power, effect‐estimate precision and ROC/AUC stability. Second, the study lacked an unexposed comparison group. Third, no environmental monitoring or biological exposure assessment was conducted; heat stress, dehydration and combustion‐related exposures were not demonstrated in this cohort and are considered only plausible occupational hypotheses supported by previous literature. Fourth, urinary KIM‐1 and NGAL were analysed as absolute concentrations without adjustment for urine dilution. Urinary creatinine was not measured, preventing creatinine‐based normalisation of both biomarkers. Urine specific gravity was also unavailable for alternative adjustment. Variations in hydration status may have influenced urinary biomarker concentrations and increased measurement variability. These limitations may have weakened the observed associations with eGFR and reduced discriminatory performance. Fifth, eGFR was measured at a single time point, and albuminuria was unavailable; the study‐defined eGFR < 90 category cannot be interpreted as CKD or confirmed kidney impairment. Sixth, several potential confounders, including blood pressure, diabetes, body mass index, medication use, hydration status, smoking, and workload, were not available for adjusted analysis. A serum creatinine value of 0.15 mg/dL was confirmed in the laboratory report; sensitivity analysis excluding this participant yielded similar AUCs and did not materially change the exploratory interpretation.

### 4.2 Implications for Future Research

Future studies should recruit a larger sample with an appropriate comparison group from non‐tofu occupations or other relevant worker populations. Exposure assessment should include wet bulb globe temperature or equivalent heat‐stress indices, workload estimation, fluid intake, urine specific gravity or osmolality, and potential nephrotoxic exposures such as metals or combustion‐related pollutants. Kidney assessment should include serum creatinine, cystatin C, urine albumin‐creatinine ratio, urinary creatinine‐normalised KIM‐1 and NGAL, and repeated measurements over time. A longitudinal design would be preferable for determining whether tubular injury biomarkers predict subsequent kidney function decline among tofu production workers.

## 5 Conclusion

In this pilot study, serum and urinary KIM‐1 and NGAL poorly discriminated the study‐defined eGFR < 90 category. The AUC estimates of 0.327–0.416, lower biomarker medians in the eGFR < 90 group, and weak correlation estimates indicate that these biomarkers should not be interpreted as stand‐alone diagnostic tools for the study‐defined eGFR < 90 category in this dataset. These preliminary findings do not establish the broader diagnostic value of KIM‐1 or NGAL as tubular injury biomarkers. Larger exposure‐informed studies should evaluate these biomarkers against injury‐specific kidney outcomes, with urinary creatinine‐normalised biomarkers, albuminuria, repeated kidney function measurements and adequate adjustment for clinical and occupational confounders.

## Author Contributions

**Putri Ayuni Alayyannur:** conceptualization; writing – original draft; visualization; formal analysis; data curation. **Yustinus Denny Ardyanto Wahyudiono:** conceptualization; supervision. **Addien Nadira:** writing – review and editing; investigation; conceptualization; validation. **Ritwan Agus Prasetyo:** visualization; formal analysis; data curation; resources. **Edza Aria Wikurendra:** writing – review and editing; visualization; validation; formal analysis; supervision.

## Disclosure

All authors have read and approved the final version of the manuscript. Edza Aria Wikurendra, the corresponding author, had full access to all of the data in this study and takes complete responsibility for the integrity of the data and the accuracy of the data analysis.

## Ethics Statement

The study protocol was approved by the Research and Public Health Service Ethics Committee, Faculty of Public Health, Universitas Indonesia (Ethical Clearance No. Ket‐550/UN2.F10.D11/PPM.00.02/2022).

## Conflicts of Interest

The authors declare no conflicts of interest.

## Transparency Statement

Edza Aria Wikurendra, the manuscript guarantor, affirms that this manuscript is an honest, accurate, and transparent account of the study being reported; that no important aspects of the study have been omitted; and that any discrepancies from the study as planned (and, if relevant, registered) have been explained.

## Data Availability

The datasets generated and analysed during the current study are available from the corresponding author upon reasonable request. Access is subject to institutional and ethical approval requirements to protect participant confidentiality. No analysis code or additional research materials are publicly available; the statistical procedures and laboratory methods required to interpret the results are described in the Methods section.
